# Soybean β-Conglycinin Induces Inflammation and Oxidation and Causes Dysfunction of Intestinal Digestion and Absorption in Fish

**DOI:** 10.1371/journal.pone.0058115

**Published:** 2013-03-08

**Authors:** Jin-Xiu Zhang, Lin-Ying Guo, Lin Feng, Wei-Dan Jiang, Sheng-Yao Kuang, Yang Liu, Kai Hu, Jun Jiang, Shu-Hong Li, Ling Tang, Xiao-Qiu Zhou

**Affiliations:** 1 Animal Nutrition Institute, Sichuan Agricultural University, Chengdu, Sichuan, China; 2 Fish Nutrition and Safety Production University Key Laboratory of Sichuan Province, Sichuan Agricultural University, Chengdu, Sichuan, China; 3 Key Laboratory for Animal Disease-Resistance Nutrition of China Ministry of Education, Sichuan Agricultural University, Chengdu, Sichuan, China; 4 Animal Nutrition Institute, Sichuan Academy of Animal Science, Chengdu, China; The University of Plymouth, United Kingdom

## Abstract

β-conglycinin has been identified as one of the major feed allergens. However, studies of β-conglycinin on fish are scarce. This study investigated the effects of β-conglycinin on the growth, digestive and absorptive ability, inflammatory response, oxidative status and gene expression of juvenile Jian carp (*Cyprinus carpio* var. Jian) *in vivo* and their enterocytes *in vitro*. The results indicated that the specific growth rate (SGR), feed intake, and feed efficiency were reduced by β-conglycinin. In addition, activities of trypsin, chymotrypsin, lipase, creatine kinase, Na^+^,K^+^-ATPase and alkaline phosphatase in the intestine showed similar tendencies. The protein content of the hepatopancreas and intestines, and the weight and length of the intestines were all reduced by β-conglycinin. β-conglycinin increased lipid and protein oxidation in the detected tissues and cells. However, β-conglycinin decreased superoxide dismutase (SOD), catalase (CAT), glutathione-*S*-transferase (GST), glutathione peroxidase (GPx) and glutathione reductase (GR) activities and glutathione (GSH) content in the intestine and enterocytes. Similar antioxidant activity in the hepatopancreas was observed, except for GST. The expression of target of rapamycin (TOR) gene was reduced by β-conglycinin. Furthermore, mRNA levels of interleukin-8 (IL-8), tumor necrosis factor-α (TNF-α), and transforming growth factor-β (TGF-β) genes were increased by β-conglycinin. However, β-conglycinin increased CuZnSOD, MnSOD, CAT, and GPx1b gene expression. In conclusion, this study indicates that β-conglycinin induces inflammation and oxidation, and causes dysfunction of intestinal digestion and absorption in fish, and finally reduces fish growth. The results of this study provide some information to the mechanism of β-conglycinin-induced negative effects.

## Introduction

Soybean meal (SBM) is an excellent plant protein source for fish feed [1]. However, high levels of soybean protein caused a poor growth rate in juvenile Jian carp (*Cyprinus carpio* var. Jian) [2] and juvenile tilapia (*Oreochromis niloticus×O. aureus*) [3]. In addition, SBM has been shown to decrease intestinal protease activity in juvenile tilapia [3] and reduce intestinal alkaline phosphatase (AKP) activity in Jian carp [4]. These negative influences were partly due to the anti-nutritional factors present in SBM, such as β-conglycinin, glycinin, and trypsin inhibitor [5]. Among these anti-nutrients, β-conglycinin was suggested as being one of the key anti-nutritional factors [6]. A recent study has reported that β-conglycinin reduced weight gain in pigs [7]. However, little attention has been given to the potential negative effects of β-conglycinin on the growth, digestive and absorptive ability of fish.

The reductions in digestive and absorptive ability may be associated with a diminished growth of the intestine; it has been reported that β-conglycinin decreased the villus height in piglet intestines [8]. Protein not only provides architectural support for cells but also serves vital roles in maintaining their function and survival, and protein deposition is a result of protein synthesis and degradation [9]. It was demonstrated that the target of rapamycin (TOR) signaling pathway plays an important role in protein synthesis and degradation in terrestrial animals, and the eIF4E-binding protein (4E-BP) is one of the major downstream targets of TOR protein [10]. Our laboratory was the first to clone the cDNA of TOR (GenBank accession no. FJ899680) and 4E-BP2 (GenBank accession no. HQ010440) of Jian carp and to show that the expressions of these two genes were influenced by choline [11] and arginine [12]. Accordingly, further investigation is warranted into whether β-conglycinin causes poor growth of digestive organs via changing the TOR and 4E-BP gene expression.

The decreased growth, development and function of the digestive organs may be partly due to the fact that β-conglycinin damages the integrity of the intestine. Previous studies have shown that β-conglycinin causes pathological disruption in the intestines of rats [13] and piglets [8]. Enterocytes are the primary cells lining the intestine [14], and increasing levels of β-conglycinin damaged the integrity of mouse enterocytes, leading to LDH releasing into the culture medium [15]. Thus, we hypothesize that β-conglycinin may also injure the intestine and its enterocytes in fish, a theory that requires further investigation.

It has been shown in pigs that the intestinal injury caused by β-conglycinin results from the effect of this allergen as an inducer of stress [16]. Allergies usually cause inflammatory disorders [17]; for example, it has been reported that β-conglycinin promoted the secretion of inflammatory cytokines (IL-8) in mouse enterocytes [15]. In addition, transforming growth factor β (TGF-β) was identified as an important factor in the prevention of intestinal mucosal inflammation in humans and mice [18]. A study of Atlantic salmon (*Salmo salar* L.) showed that the TGF-β gene level was significantly decreased by dietary SBM [19]. However, little attention has been given to the potential negative effects of β-conglycinin on these inflammatory factors in fish.

Inflammation is always accompanied by lipid oxidation, the end products of which are generated during inflammation in mice [20]. Thus, it appears that β-conglycinin-induced intestinal damage may be involved in lipid peroxidation. In addition, lipid peroxidation may bring about protein damage due to its end products [21]. Protein carbonyl content is the most widely used biomarker for oxidative damage to proteins and reflects cellular damage [22]. Like other organisms, fish combat elevated levels of oxidative stress with protective enzymes, including superoxide dismutase (SOD), catalase (CAT) and glutathione-dependent enzymes [21]. However, studies referring to the effects of β-conglycinin on the antioxidant system are scarce. To our knowledge, only this study has examined the activities and mRNA levels of antioxidant enzymes in assessing the antioxidant defenses challenged with β-conglycinin.

To this context, we hypothesize that β-conglycinin may reduce fish growth through damaging digestive organ which was in part attributed to antioxidant disturbance. Thus, efficient antioxidant becomes of great important. Glutamine (Gln), a conditionally essential amino acid, appears to be a key nutrient for the gut in mammalian [23]. Our laboratory has demonstrated that dietary Gln supplementation increased the intestinal weight and alkaline phosphosphate activity, and thus improved fish intestinal structure and function in Jian carp [24]. Furthermore, Gln serves as a metabolic precursor for glutathione (GSH) which can efficiently reduce ROS [25], [26]. It can protect fish enterocytes against H_2_O_2_-induced oxidative damage [27]. Thus, we set up a treatment that administrated Gln along with β-conglycinin to study the hypothesis that Gln can protect fish against β-conglycinin toxicity.

Together, this study investigated the hypothesis that β-conglycinin might decrease the growth of fish via dysfunction of digestion and absorption which was in part attributed to the digestive organ damage induced by β-conglycinin-mediated inflammation and oxidative stress. To our knowledge, this study provides the first insight into the underlying mechanism by which β-conglycinin decreases digestive and absorptive ability and damages intestinal integrity in fish. The potential protective effect of Gln against β-conglycinin toxicity was also investigated.

## Materials and Methods

### Growth Trial (*in vivo*)

Purified β-conglycinin was kindly donated by the China Agricultural University (Patent No. 200410029589·4, China) and was analyzed following the method of [8] to be 80% pure. The test diets are presented in Table S1. The control diet, devoid of soybean protein, contained fish meal, gelatin and casein as the dietary protein sources. β-conglycinin (80 g β-conglycinin/kg diet) was substituted for the casein in the corresponding experimental diets, following the method of [28]. β-conglycinin accounts for about 30% of the total soybean proteins [29]. Our previous study found that diet with 517.8 g/kg of dehulled soybean meal (CP = 47%) (approximately 73 g/kg of β-conglycinin) significantly depressed the growth, feed efficiency, intestinal weight, length, length index and folds height and the intestinal AKP and γ-GT activities in Jian carp [2], [4]. Therefore, to investigate the toxic effects and potential toxic mechanisms of β-conglycinin in fish, 80.0 g/kg of β-conglycinin was used in this study, which was proved to be enough to induce toxicity in fish by a preliminary experiment (unpublished data). β-conglycinin with 12.0 g/kg of the Gln group was established as a positive control. The concentration of Gln was the optimal dose for the growth of Jian carp [24]. All diets were made isonitrogenous with the addition of appropriate amounts of glycine according to the method of [24]. Lysine, methionine, threonine, pyridoxine, pantothenic acid, inositol, thiamin, riboflavin, zinc and iron were prepared to meet the nutrient requirements of juvenile Jian carp according to our laboratory studies [30]–[39]. The levels of other nutrients met the requirements of common carp (*Cyprinus carpio* L.) according to the NRC (1993) [40]. Diets were prepared by thoroughly mixing all the ingredients. Distilled water (approximately 40%, w/w) was added to the premix dry ingredients and further thoroughly mixed. Slow sinking pellets were wet-extruded through a laboratory-scale, single screw extruder. The machine was continually cooled by a cooling wet paper tower. Thus, the temperature of wet-extruded pellets was controlled to be between 55 and 65°C. The noodle-like diets were immediately freeze-dried at −55°C. After freeze-drying, the pellets were stored at −20°C until feeding according to the method described by [41].

The Animal Care and Use Committee of Sichuan Agricultural University approved all experimental procedures. Juvenile Jian carp (*Cyprinus carpio* var. Jian) purchased from Tong Wei Hatchery (Sichuan, China) were used in this experiment. Fish were acclimatized to the experimental environment for 4 weeks, and were fed with a carp feed (without soybean protein, prepared in our laboratory, crude protein = 34%, crude lipid = 4.6%). Four hundred and fifty fish, with a mean initial weight of 5.36±0.02 g, were randomly assigned to each of 9 experimental aquaria (90 L×30 W×40 H cm). Aquaria were supplied with flow-through water at a rate of 1.2 L/min; water was drained through biofilters to remove solid substances and reduce ammonia concentration. Water temperature was maintained at 23±1°C. For the feeding trial, each of the diets was fed to a triplicate of fish six times per day for the first 4 weeks and four times per day from the 5^th^ to 6^th^ week, a feeding rhythm that was established in previous study [42]. The fish were fed their respective diets to apparent satiation. Uneaten feed was removed by siphoning thirty minutes after feeding, dried and weighed to calculate feed intake.

Fish from each aquarium were counted and weighed at the beginning and end of the experiment. At the end of the experiment, fish were anaesthetized in benzocaine bath (50 mg/L) 12 h after the last feeding as described by [43]; the intestines and hepatopancreas were removed quickly, weighed, frozen in liquid nitrogen and stored at −70°C until analysis.

### *In vitro* Experiment

The procedures for cell isolates and cultures were based on those of [44] and [45], with slight modifications. Healthy Jian carp were maintained for approximately 24 h without feeding and were killed by decapitation. The intestines were rapidly removed from the carcass, opened and rinsed with Hanks Balanced Salt Solution (HBSS). The enterocytes were isolated by enzymatic dissociation. Cells were suspended in Dulbecco’s Modified Eagle’s Media (DMEM) and washed with the same medium 5 times to remove enzymes. Isolated enterocytes were plated in high-glucose DMEM supplemented with 10% FBS, 0.02 mg transferring/mL (Sigma, St. Louis, MO, USA), 0.01 mg insulin/mL (Sigma, St. Louis, MO, USA), 100 IU benzyl penicillin/mL (Sigma, St. Louis, MO, USA) and 100 µg streptomycin sulfate/mL (Sigma, St. Louis, MO, USA). The cells (2×10^3^ cells per well) were seeded in 24-well culture plates (Falcon, Franklin Lake, NJ, USA) that had been previously coated with collagen (Sigma, St. Louis, MO, USA), as previously described by [46]. Cultures were kept at 26±0.5°C. The β-conglycinin treatment was performed by adding fresh medium containing 0, 0.25, 0.6, 0.9, 1.2 and 2.5 mg/mL for 36 h. Four replicate wells were prepared for each concentration of β-conglycinin (n = 4).

The second *in vitro* trial was based on the first *in vitro* trial and was performed similarly, except for the concentrations of β-conglycinin (0, 1.2, 2.5, 3.5, 4.5 and 5.5 mg/mL).

### Analysis and Measurement Methods

#### Investigated indexes

The *in vivo* experiment investigated the hepatopancreas weight (HW), protein content (HPC), hepatopancreas somatic index (HSI) and intestinal weight (IW), protein content (IPC), length (IL), intestinal length index (ILI), intestinal somatic index (ISI) and folds height. In addition, digestive ability (intestinal trypsin, chymotrypsin, lipase and amylase activities) and absorptive function (creatinkinase, Na^+^,K^+^-ATPase, alkaline phosphatase and gamma-glutamyl transpeptidase (γ-GT) in the proximal (PI), mid (MI) and distal intestine (DI)) were studied. Furthermore, malondialdehyde (MDA), protein carbonyls (PC), superoxide dismutase (SOD), catalase (CAT), glutathione-*S*-transferase (GST), glutathione peroxidase (GPx) and glutathione reductase (GR) activities and glutathione (GSH) content in the hepatopancreas and intestine were also investigated. Finally, gene expression of TOR and 4E-BP in the PI, MI and DI and interleukin-1 (IL-1), IL-8, tumor necrosis factor-α (TNF-α) and TGF-β genes in the distal intestine (DI) as well as the whole intestinal GPx1a, GPx1b, GR, CuZnSOD, MnSOD and CAT were investigated.

The first *in vitro* trial investigated the cell viability (MTT), protein retention (PR), glutamate oxaloacetate transaminase (GOT) in enterocytes and LDH, GOT and glutamate pyruvate transaminase (GPT) activities in culture medium. The second *in vitro* trail investigated the oxidative damage indexes (including LDH and MDA in medium and cellular PC) and the cellular functional indexes (AKP) as well as cell antioxidant indexes (including SOD, CAT, GPx, GST, GR and GSH).

#### Digestive and absorptive analysis

At the end of the growth trail, the intestines of 5 fish from each aquarium were sampled and fixed with formalin (10%), sectioned and stained with H-E stain for histological analysis (height of intestinal folds) according to the method described by Lin and Zhou [24].

Samples of the intestine and hepatopancreas were each homogenized in 10 volumes (w/v) of ice-cold physiological saline solution and centrifuged at 6,000×***g*** for 20 min at 4°C, respectively. After centrifugation, the supernatant was used for determination of trypsin [47], chymotrypsin [47], lipase [48], amylase [49], alkaline phosphatase (AKP) [50] and Na^+^, K^+^-ATPase activities [51]. Gamma-glutamyl transpeptidase (γ-GT) activities in the intestine were determined by using a γ-GT Assay Kit (Sigma, St Louis, MO). Protein content was determined following the Bradford method [52].

#### MTT cell viability assay

Cell proliferation was determined by assaying the reduction of 3-(4,5-dimethylthiazol-2-yl)-2,5-diphenyltetrazolium bromide (MTT) (Sigma) to formazan, following the method described by [53]. Briefly, at the end of the experiment, the medium was removed and 500 µL of MTT working solution was added to the cultures. After incubation for 4 h, the MTT working solution was removed and replaced with 500 µL of dimethyl sulphoxide (Sigma) to dissolve the formazan precipitates. The amount of formazan was determined by measuring the optical density (OD) at 595 nm on a plate reader (Thermo Labsystems Oy, Helsinki, Finland).

#### Cell injury analysis

The amount of LDH released by the cells was measured following the method of [54]. Lipid peroxidation was analyzed as described by [55] and measured in terms of malondialdehyde (MDA) equivalents using the thiobarbituric acid (TBA) reaction. In brief, samples were mixed with trichloroacetic acid and centrifuged. Then, TBA was added to the supernatant. The mixture was heated in water at 95°C for 40 minutes. MDA forms a red adduct with TBA, which has an absorbance of 532 nm. The cellular protein carbonyl (PC) content was determined according to the method described by [22], with a minor modification using the 2,4-dinitrophenylhydrazine (DNPH) reagent. The carbonyl content was calculated from the peak absorbance at 340 nm, using an absorption coefficient of 22,000/M/cm.

#### Antioxidant enzyme activities and GSH content

The abilities of the anti-superoxide anion (ASA) and the anti-hydroxyl radical (AHR) (OH-scavenging ability) were determined following the instructions of the specific kits (Jiancheng Bioengineering Ltd., Nanjing, China). Briefly, superoxide radicals (O_2_^−^) were generated by the action of xanthine and xanthine oxidase. With the addition of an electron acceptor, a coloration reaction (absorbance at 550 nm) was developed using the gross reagent. Vitamin C was used as the standard agent. AHR was then assayed based on the Fenton reaction (Fe^2+^+H_2_O_2_→Fe^3+^+OH^−^+•OH). A coloration reaction (absorbance at 550 nm) was also developed using the gross reagent. CAT activities were measured, as have previously described [56]. The superoxide dismutase (SOD) and glutathione peroxidase (GPx) activities were assayed as described by [55]. The glutathione-*S*-transferase (GST) activity was measured by monitoring the formation of an adduct between GSH and 1-chloro-2,4-dinitrobenzene (CDNB) [57]. GSH content was determined by the method described by [58] with a minor modification.

#### Analysis of gene expression

The total RNA of samples was extracted using RNAiso Plus (TaKaRa Biotechnology, Dalian Co. Ltd., China, D9108B), according to the manufacturer’s instructions. RNA quantity and quality were assessed by electrophoresis on 1% agarose gels and by spectrophotometry at 260 and 280 nm. Subsequently, cDNA was synthesized using a PrimeScript™ RT reagent Kit (Takara, Dalian, Liaoning, China), according to manufacturer’s instructions. Briefly, oligo dT primers (50 µM) were used to reverse transcribe respective RNAs in the presence of PrimeScript™ RT enzyme Mix I, 5×PrimeScript™ buffer, Random 6 mers (100 µM) and RNase free dH_2_O at 37°C for 15 min, following inactivation at 85°C for 5 s.

Specific primers for genes were designed with Primer Premier software (Premier Biosoft International, Palo Alto, CA, USA), according to the Jian carp sequences (except for IL-1, IL-8, TNF-α and TGF-β, the other genes were cloned and submitted to NCBI by our laboratory) (Table S2). Real-time PCRs were performed with a chromo 4™ continuous fluorescence detector (Bio-Rad Laboratories, Inc.) using a SYBR PrimeScriptTM RT-PCR Kit II, according to standard protocols. The expression levels of these genes were normalized to the expression levels of a housekeeping gene, β-actin (Table S2). Each assay was performed with 5 replications. The concentrations of the target genes were calculated based on the threshold cycle number (CT). The CT for each sample was determined by using MJ Option Monitor Software (version 3.1; Bio-Rad, Hemel Hempstead, Herts, UK). In addition, the cDNA concentration in each sample was determined according to gene-specific standard curves. Standard curves were generated for both the target and endogenous control genes based on 10-fold serial dilutions. All standard curves exhibited correlation coefficients higher than 0.99, and the corresponding real-time PCR efficiencies ranged from 0.90–1.10.

### Statistical Analysis

All data are presented as the mean ± standard deviation (SD). The data were subjected to a one-way analysis of variance (ANOVA). If significant differences were found (*P*<0.05), Duncan’s multiple range tests were used to rank the groups. TNF-α and TGF-β mRNA levels were evaluated with Student’s *t*-test. All statistical analyses were performed using the SPSS 13.0 for Windows (SPSS Inc, Chicago, IL, USA).

## Results

### *In vivo* Experiment

Initial body weight (IBW), final body weight (FBW), special growth ratio (SGR), survival rate, feed intake (FI), feed efficiency (FE), hepatopancreas weight (HW), protein content (HPC) and somatic index (HSI) and intestinal weight (IW), intestinal protein content (IPC), intestinal length (IL), intestinal length index (ILI) and intestinal somatic index (ISI) and folds height in the proximal intestine (PI), mid intestine (MI) and distal intestine (DI) are presented in Table 1. β-conglycinin alone significantly reduced FBW by approximately 15.1% compared with the control, but co-treatment with Gln completely blocked the reduction of FBW caused by β-conglycinin. Similarly, β-conglycinin alone also significantly reduced SGR compared with the unexposed control (*P<*0.05). The SGR significantly increased by co-treatment with Gln compared with that of fish exposed to β-conglycinin alone (*P<*0.05); the value was equivalent to the control. Survival was not affected by dietary treatments. The FI was lowest in fish exposed to β-conglycinin alone, and significantly recovered by co-treatment with Gln. The FE was higher in fish fed the control diet than in fish fed with β-conglycinin alone. HW was highest in fish fed the control diet, followed by fish co-fed with Gln and β-conglycinin, and was lowest in fish fed with β-conglycinin alone. HPC was higher in fish co-treated with Gln and β-conglycinin than in fish fed with β-conglycinin alone. Fish exposed to dietary β-conglycinin experienced a decrease in HSI compared with the control. There was no significant difference between the combinations and the β-conglycinin alone. IPC was lower in fish exposed to dietary β-conglycinin alone than in other groups. IW and IL were significantly reduced by dietary β-conglycinin alone compared with the unexposed control but was partly recovered by supplementation with dietary Gln. In addition, ILI was also significantly reduced by β-conglycinin alone compared with the unexposed control. However, there was no significant alteration in ISI among the treatments. The folds height in the PI was significantly reduced by dietary β-conglycinin alone compared with the unexposed control, but was partly recovered by supplementing the diet with Gln (*P<*0.05). The folds height in the MI and DI were lower in fish exposed to β-conglycinin alone than in that of the control.

**Table 1:** Initial body weight (IBW), final body weight (FBW), special growth ratio (SGR), survival rate, feed intake (FI), feed efficiency (FE), hepatopancreas weight (HW), protein content (HPC) and somatic index (HSI) and intestinal weight (IW), intestinal protein content (IPC), intestinal length (IL), intestinal length index (ILI) and intestinal somatic index (ISI) and folds height (µm) in the proximal intestine (PI), mid intestine (MI) and distal intestine (DI) of juvenile Jian carp (*Cyprinus carpio* var. Jian) exposed to dietary β-conglycinin for 42 days (*in vivo* experiment).

Indexes	Control	β-conglycinin alone	β-conglycinin plus Gln
IBW (g/fish)	5.37±0.04^a^	5.36±0.01^a^	5.36±0.01^a^
FBW (g/fish)	28.68±0.92^a^	24.34±0.86^b^	27.15±0.60^a^
SGR	3.99±0.09^a^	3.60±0.08^b^	3.86±0.05^a^
Survival rate	100.00±0.00^a^	100.00±0.00^a^	100.00±0.00^a^
FI (g/fish)	30.21±0.05^a^	26.42±0.07^c^	28.79±0.01^b^
FE	87.69±3.50^a^	81.60±3.47^b^	86.01±2.35^ab^
HW (g/fish)	1.18±0.22^a^	0.60±0.12^c^	0.73±0.24^b^
HPC	4.15±0.20^ab^	4.01±0.32^b^	4.36±0.19^a^
HSI	3.56±0.40^a^	3.07±0.34^b^	3.39±0.86^ab^
IW (g/fish)	1.10±0.20^a^	0.62±0.15^c^	0.75±0.19^b^
IPC	2.35±0.16^a^	1.99±0.13^b^	2.41±0.21^a^
IL (cm/fish)	17.93±2.34^a^	14.10±1.58^c^	15.09±2.08^b^
ILI	170.22±21.54^a^	160.42±13.04^b^	164.49±18.85^ab^
ISI	3.23±0.27^a^	3.13±0.39^a^	3.25±0.36^a^
PI folds heights	700.44±70.72^a^	536.80±22.42^c^	633.53±37.97^b^
MI folds heights	443.58±42.94^a^	378.74±23.12^b^	425.99±46.47^ab^
DI folds heights	524.14±46.60^a^	447.93±45.50^b^	482.20±34.71^ab^

Data represent means ± S.D. Values within the same row with different superscripts are significantly different (*P*<0.05).

SGR = 100× (ln final weight - ln initial weight)/number of days.

FE = 100× (g weight gain/g feed intake).

HPC = 100×(g hepatopancreas protein/g wet hepatopancreas weight).

HSI = 100×(g wet hepatopancreas weight/g wet body weight).

IPC = 100×(g intestine protein/g wet intesitine weight).

ILI = 100× (cm intestine length/cm total body length).

ISI = 100×(g wet intestine weight/g wet body weight).

Intestinal trypsin, chymotrypsin, amylase and lipase activities are presented in Table 2. The intestinal trypsin of fish fed with β-conglycinin and Gln was significantly increased compared with that of the group fed β-conglycinin alone (*P<*0.05); the value was equivalent to fish not exposed to β-conglycinin. The intestinal chymotrypsin and lipase activities reflected the same pattern in regard to intestinal trypsin activity. No significant differences were observed in intestinal amylase activities among the treatments.

**Table 2:** Intestinal digestive enzyme activity of juvenile Jian carp exposed to dietary β-conglycinin for 42 days (*in vivo* experiment).

Indexes	Control	β-conglycinin alone	β-conglycinin plus Gln
Trypsin (U/g tissue)	2.41±0.07^a^	2.14±0.24^b^	2.36±0.08^a^
Chymotrypsin (U/g tissue)	2.85±0.26^a^	1.38±0.15^b^	2.65±0.30^a^
Lipase (U/g tissue)	373.29±45.57^a^	176.56±29.76^b^	322.84±49.43^a^
Amylase (U/g tissue)	1309.52±52.42^a^	1295.24±71.52^a^	1323.81±56.18^a^

Data represent means ± S.D. of six replicates. Values within the same row with different superscripts are significantly different (*P*<0.05).

CK, Na^+^, K^+^-ATPase, AKP and γ-GT activities in the PI, MI and DI are presented in Table 3. β-conglycinin alone caused significant decreases in CK and Na^+^, K^+^-ATPase activity in all intestinal segments compared with the unexposed control but was completely recovered by a diet combined with Gln (*P<*0.05). Similarly, the AKP activity in the PI, MI and DI were lower in fish exposed to β-conglycinin alone than that of the other groups. Conversely, the gamma-glutamyl transpeptidase (γ-GT) activity in all intestinal segments was increased by β-conglycinin. Only the β-conglycinin-induced increase of γ-GT activity in the MI was partly prevented by Gln.

**Table 3:** Creatine kinase (CK, µmol of phosphorus released/g tissue/h), Na^+^, K^+^-ATPase (µmol of phosphorus released/g tissue/h), alkaline phosphatase (AKP, mmol of nitrophenol released/g tissue/h) and gamma-glutamyl transpeptidase (γ-GT, mmol of 5-amino-2-nitrobenzoate released/g tissue/min) activity in the proximal intestine (PI), mid intestine (MI) and distal intestine (DI) of juvenile Jian carp exposed to dietary β-conglycinin for 42 days (*in vivo* experiment).

Indexes	Intestines	Control	β-conglycinin alone	β-conglycinin plus Gln
**CK**				
	PI	47.14±4.60^a^	41.62±2.17^b^	46.52±3.90^a^
	MI	55.77±5.74^a^	43.36±5.63^b^	50.68±4.66^a^
	DI	22.38±2.37^b^	16.60±2.49^c^	30.94±3.12^a^
**Na^+^, K^+^-ATPase**				
	PI	289.06±31.54^a^	237.63±42.39^b^	298.83±35.00^a^
	MI	220.70±30.23^a^	158.85±24.29^b^	195.31±16.01^a^
	DI	207.68±36.26^a^	144.53±54.07^b^	203.13±45.29^a^
**AKP**				
	PI	26.23±3.19^a^	21.44±2.52^b^	25.82±3.38^a^
	MI	17.28±2.09^a^	13.43±2.47^b^	16.53±1.83^a^
	DI	3.30±0.24^a^	2.83±0.30^b^	3.28±0.32^a^
**γ-GT**				
	PI	38.37±4.85^b^	89.77±22.69^a^	82.91±14.52^a^
	MI	65.47±9.02^c^	214.30±37.74^a^	134.42±17.60^b^
	DI	80.81±11.07^b^	180.23±26.50^a^	160.82±23.32^a^

Data represent means ± S.D. of six replicates. Values within the same row with different superscripts are significantly different (*P*<0.05).

The oxidative status of the hepatopancreas and intestines and their antioxidant levels are presented in Table 4. The MDA and PC content in the hepatopancreas and intestine were lower in fish fed the control diet than in the other groups (*P<*0.05). There was no change between the β-conglycinin group and the Gln plus β-conglycinin group. In addition, except for GST, the antioxidants (SOD, CAT, GPx and GR activities and GSH content) in the hepatopancreas and intestine were reduced by β-conglycinin compared with the unexposed control. The decreases in these antioxidants were completely recovered by supplementation with dietary Gln (*P<*0.05). There was no significant change in GST activity in the hepatopancreas among the treatments. In addition, GST activity in the intestine was reduced by β-conglycinin (*P<*0.05), but no significant difference was observed between the β-conglycinin alone group and the Gln plus β-conglycinin group.

**Table 4:** Oxidative status (malondialdehyde (MDA, nmol/mg protein), protein carbonyl content (PC, nmol/mg protein) and antioxidative enzyme activities (superoxide dismutase (SOD, U/mg protein), catalase (CAT, U/mg protein), glutathione-*S*-transferase (GST, U/mg protein), glutathione peroxidase (GPx, U/mg protein), glutathione reducase (GR, U/g protein) activities and glutathione (GSH, mg/g protein) content) in the hepatopancreas and intestine of juvenile Jian carp exposed to dietary β-conglycinin for 42 days (*in vivo* experiment).

Organs	Indexes	Control	β-conglycinin alone	β-conglycinin plus Gln
**Hepatopancreas**				
	MDA	1.12±0.09^b^	1.38±0.12^a^	1.29±0.08^a^
	PC	0.71±0.13^b^	1.08±0.14^a^	0.96±0.15^a^
	SOD	41.66±9.02^a^	21.25±5.67^b^	28.91±5.58^b^
	CAT	11.40±1.36^a^	8.78±1.14^b^	10.76±1.65^a^
	GST	32.49±5.13^a^	27.80±4.31^a^	29.36±3.79^a^
	GPx	404.0±31.3^a^	313.7±51.0^b^	358.6±43.0^ab^
	GR	39.56±7.13^a^	25.03±4.48^b^	34.54±7.57^a^
	GSH	24.31±2.66^a^	19.30±1.75^b^	20.97±2.95^b^
**Intestine**				
	MDA	2.83±0.31^b^	3.58±0.40^a^	3.42±0.18^a^
	PC	1.16±0.18^b^	1.72±0.38^a^	1.56±0.16^a^
	SOD	46.13±7.69^a^	30.61±4.58^b^	41.69±2.76^a^
	CAT	11.03±0.94^a^	8.90±1.09^b^	10.13±0.82^a^
	GST	83.42±10.46^a^	51.98±7.93^b^	62.84±12.21^b^
	GPx	174.9±23.7^a^	143.1±13.2^b^	170.8±26.1^a^
	GR	91.09±12.86^a^	67.48±12.26^b^	82.00±11.08^ab^
	GSH	38.21±2.99^a^	29.82±2.78^b^	35.49±6.85^ab^

Data represent means ± S.D. of six replicates. Values within the same row with different superscripts are significantly different (*P*<0.05).

The TOR and 4E-BP gene levels in the PI, MI and DI are presented in Table 5. β-conglycinin significantly reduced the levels of TOR mRNA in the PI and MI (*P<*0.05). However, Gln was found to completely block the reductions in TOR expression caused by β-conglycinin in the PI and partly block them in the MI. The TOR gene expression levels in the DI were not significantly different among the three groups, only showing a tendency to decrease with β-conglycinin. The results indicated that β-conglycinin caused an increase in 4E-BP gene expression in the DI, which could partly be reversed by Gln. However, the levels of 4E-BP gene expression in the PI and MI showed no significant differences among the three groups.

**Table 5:** Relative expression of TOR and 4E-BP genes in the proximal intestine (PI), mid intestine (MI) and distal intestine (DI) of juvenile Jian carp exposed to dietary β-conglycinin for 42 days (*in vivo* experiment).

Genes	Intestines	Control	β-conglycinin alone	β-conglycinin plus Gln
**TOR**				
	PI	0.012±0.0032^a^	0.0075±0.0017^b^	0.012±0.0022^a^
	MI	0.033±0.0047^a^	0.0086±0.0014^c^	0.016±0.0026^b^
	DI	0.0617±0.0128^a^	0.0538±0.0152^a^	0.0573±0.0178^a^
**4E-BP**				
	PI	0.060±0.018^a^	0.082±0.025^a^	0.091±0.025^a^
	MI	0.0029±0.0005^a^	0.0024±0.0004^a^	0.0026±0.0008^a^
	DI	0.0041±0.0012^c^	0.015±0.0036^a^	0.011±0.0033^b^

Data represent means ± S.D. of five replicates. Values within the same row with different superscripts are significantly different (*P*<0.05).

**Figure 1:**
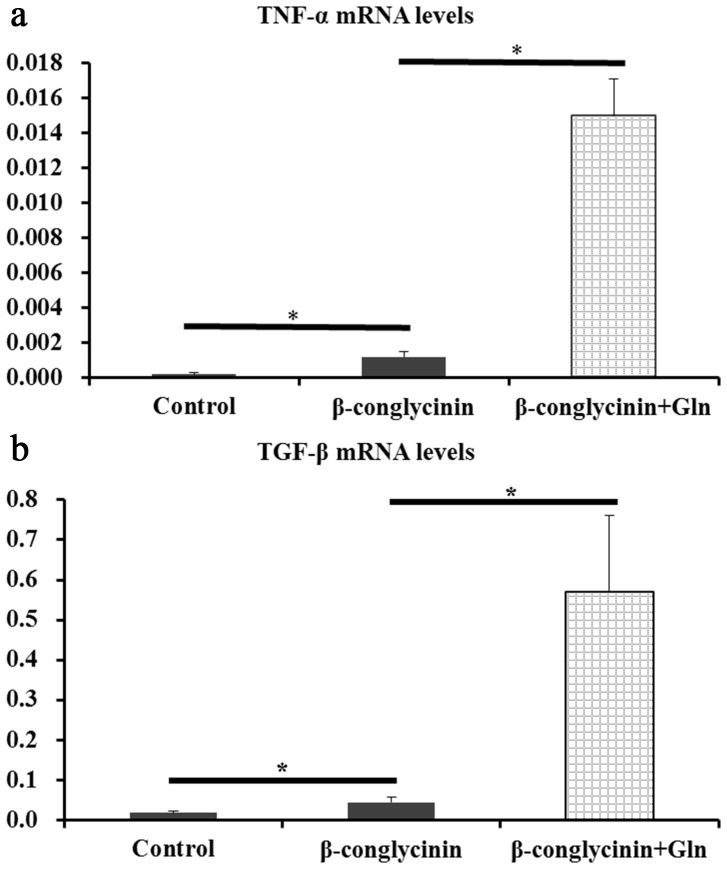
Student’s *t-*test of TNF-α (a) and TGF-β mRNA (b) levels. Data represent means ± S.D. of five replicates. Values with * are significantly different (*P*<0.05).

To investigate the effects of β-conglycinin on the intestinal inflammatory response in fish, the expressions of IL-1, IL-8, TNF-α and TGF-β genes in the DI were determined (Table 6). The highest levels of IL-1, TNF-α and TGF-β genes were observed in the β-conglycinin with Gln group (*P<*0.05). The relative expressions of IL-8 were reduced by β-conglycinin (*P<*0.05). However, the mean values of TNF-α and TGF-β mRNA levels among the three groups are largely different. The TNF-α and TGF-β were not significantly affected by β-conglycinin treatment when Duncan’s multiple range tests were used (Table 6). However, TNF-α (Figure 1a) and TGF-β (Figure 1b) were significantly increased by β-conglycinin treatment when Student’s *t* test was used.

**Table 6:** Relative expression of interleukin-1 (IL-1), IL-8, tumor necrosis factor-α (TNF-α) and transforming growth factor β (TGF-β) genes in the distal intestine (DI) of juvenile Jian carp (*Cyprinus carpio* var. Jian) exposed to dietary β-conglycinin for 42 days (*in vivo* experiment).

Genes	Control	β-conglycinin alone	β-conglycinin plus Gln
IL-1	0.0021±0.0004^b^	0.0014±0.0009^b^	0.0036±0.0009^a^
IL-8	0.0020±0.0004^a^	0.00060±0.0002^b^	0.00040±0.0001^b^
TNF-α	0.0002±0.0001^b^	0.0012±0.0003^b^	0.015±0.0021^a^
TGF-β	0.019±0.0045^b^	0.045±0.012^b^	0.57±0.19^a^

Data represent means ± S.D. of five replicates. Values within the same row with different superscripts are significantly different (*P*<0.05).

Table 7 shows the relative expressions of CuZnSOD, MnSOD, CAT, GPx1a, GPx1b and GR genes in the whole intestine of fish exposed to dietary β-conglycinin. The expressions of CuZnSOD, MnSOD, CAT and GPx1b genes were all increased by β-conglycinin (*P<*0.05). However, only MnSOD gene expression was completely recovered with Gln supplementation. The levels of GPx1a and GR gene expressions showed similar trends, but there were no significant differences among the three groups.

**Table 7:** Relative expression of CuZnSOD, MnSOD, CAT, GPx1a, GPx1b and GR genes in the whole intestine of juvenile Jian carp (*Cyprinus carpio* var. Jian) exposed to dietary β-conglycinin for 42 days (*in vivo* experiment).

Genes	Control	β-conglycinin alone	β-conglycinin plus Gln
CuZnSOD	0.026±0.0086^b^	0.037±0.0034^a^	0.042±0.011^a^
MnSOD	0.0064±0.0020^b^	0.013±0.0038^a^	0.0065±0.0016^b^
CAT	0.054±0.0067^b^	0.095±0.024^a^	0.075±0.017^ab^
GPx1a	0.32±0.097^a^	0.36±0.047^a^	0.34±0.092^a^
GPx1b	0.020±0.0055^b^	0.057±0.015^a^	0.050±0.016^a^
GR	0.019±0.0058^a^	0.020±0.0061^a^	0.014±0.0041^a^

Data represent means ± S.D. of five replicates. Values within the same row with different superscripts are significantly different (*P*<0.05).

### *In vitro* Experiment

#### First *in vitro* trial

The effects of β-conglycinin on MTT OD, protein retention (PR) and glutamic-oxaloacetic transaminase (GOT) activity in enterocytes, and the activities of LDH, GOT and GPT in medium are presented in Table 8. MTT OD was significantly reduced with higher levels of β-conglycinin, and the lowest MTT OD was observed in cells treated with 2.5 mg/mL of β-conglycinin (*P<*0.05). Cellular PR was highest in cells incubated in the control medium, and lowest in cells exposed to 2.5 mg/mL of β-conglycinin. Cellular GOT activity was lower in cells exposed to 1.2 and 2.5 mg/mL of β-conglycinin than cells in the other groups. LDH in medium was significantly higher in 2.5 mg/mL of β-conglycinin than LDH in the control group. GOT activities in medium were higher in 1.2 and 2.5 mg/mL of β-conglycinin than in the other treatments. GPT activities in medium were highest in 2.5 mg/mL of β-conglycinin, followed by 1.2 and 0.9 mg/mL in the β-conglycinin groups, and lowest in the 0–0.6 mg/mL of β-conglycinin groups. It should be noted that the cellular PR, cellular GOT, and LDH, GOT and GPT in medium, were not significantly different among the treatments of 0, 0.25, 0.6 and 0.9 mg/mL of β-conglycinin. Thus, we deleted those doses in the second *in vitro* trial.

**Table 8:** Effects of β-conglycinin (mg/mL) on MTT OD, protein retention (PR, µg) and glutamic-oxaloacetic transaminase (GOT, U/mg protein) activity in enterocytes and the activities of lactate dehydrogenase (LDH, U/L), GOT (U/L) and glutamic-pyruvic transaminase (GPT, U/L) in medium (the first *in vitro* experiment).

β-conglycinin	MTT OD	PR	GOT	LDH	GOT	GPT
0.00	0.115±0.002^a^	53.5±2.6^a^	20.0±3.7^a^	243±7.6^b^	6.93±0.53^b^	6.51±0.92^c^
0.25	0.108±0.002^b^	44.3±15.4^ab^	17.1±4.5^a^	255±16^ab^	7.11±1.05^b^	6.75±0.71^c^
0.60	0.104±0.002^bc^	43.9±5.7^ab^	17.6±6.6^a^	250±23^ab^	7.35±1.09^b^	6.69±0.63^c^
0.90	0.103±0.002^c^	40.0±4.6^ab^	14.4±10^a^	271±37^ab^	7.47±0.20^b^	7.59±0.82^bc^
1.20	0.101±0.002^c^	43.6±4.7^ab^	12.6±4.6^b^	262±13^ab^	9.80±1.15^a^	8.25±0.50^b^
2.50	0.093±0.007^d^	35.4±10.7^b^	12.0±3.4^b^	280±15^a^	10.72±0.42^a^	10.24±0.46^a^

Data represent means ± S.D. of four replicates. Values within the same column with different superscripts are significantly different (*P*<0.05).

#### Second *in vitro* trial

AKP and PC in cells and LDH and MDA in medium are presented in Table 9. AKP activity in cells decreased with higher levels of β-conglycinin, up to 4.5 mg/mL (*P*<0.05), with no difference shown between 4.5 and 5.5 mg/mL (*P*>0.05). LDH and MDA content in medium significantly increased with increasing levels of β-conglycinin, up to 4.5 mg/mL (*P*<0.05), and no differences were found with a further increase in β-conglycinin concentration (*P*>0.05). Cellular PC increased with higher levels of β-conglycinin, up to 4.5 mg/mL (*P*<0.05), with no difference found between 4.5 and 5.5 mg/mL of β-conglycinin (*P*>0.05).

**Table 9:** Effects of β-conglycinin (mg/mL) on the activity of alkaline phosphatase (AKP, U/g protein) in enterocytes, lactate dehydrogenase (LDH, U/L) in medium, content of malondialdehyde (MDA, nmol/mL) in medium and protein carbonyls (PC, nmol/mg protein) in enterocytes (the second *in vitro* experiment).

β-conglycinin	AKP in cell	LDH	MDA	PC in cell
0.00	1.97±0.18^a^	9.13±2.03^d^	1.30±0.05^d^	0.50±0.08^a^
1.20	1.79±0.09^ab^	10.14±2.34^cd^	1.39±0.12^d^	0.53±0.09^a^
2.50	1.59±0.14^bc^	18.26±4.06^bc^	1.58±0.03^c^	0.66±0.09^b^
3.50	1.56±0.17^c^	24.34±6.62^ab^	1.67±0.16^bc^	0.85±0.07^c^
4.50	1.22±0.17^d^	30.43±7.77^a^	1.72±0.09^a^	1.13±0.09^cd^
5.50	1.02±0.09^d^	30.42±7.72^a^	1.81±0.02^a^	1.15±0.14^d^

Data represent means ± S.D. of four replicates. Values within the same column with different superscripts are significantly different (*P*<0.05).

Cellular SOD, CAT, GPx, GST and GR activities and GSH content are shown in Table 10. SOD activities decreased with higher levels of β-conglycinin, up to 3.5 mg/mL (*P*<0.05), and plateaued thereafter (*P*>0.05). A dose-dependent decrease in CAT activities was observed in the presence of β-conglycinin and was lowest when β-conglycinin reached 5.5 mg/mL (*P*<0.05). GPx activities were highest in the group containing no β-conglycinin, followed by 1.2–4.5 mg/mL, with GPx being lowest in the presence of 5.5 mg/mL of β-conglycinin (*P*<0.05). GST activity in cells treated with 5.5 mg/mL of β-conglycinin significantly decreased by approximately 34.4%, compared with the cells incubated with no β-conglycinin (*P*<0.05). GR activity and GSH content also decreased with higher levels of β-conglycinin, up to 2.5 mg/mL (*P*<0.05), and no differences were found with a further increase in β-conglycinin concentrations (*P*>0.05).

**Table 10:** Activities of SOD (U/mg protein), CAT (U/mg protein), GPx (U/mg protein), GST (U/mg protein), GR (U/g protein) and content of GSH (mg/g protein) in enterocytes exposed to β-conglycinin (mg/mL) (the second *in vitro* experiment).

β-conglycinin	SOD	CAT	GPx	GST	GR	GSH
0.00	12.2±1.3^a^	1.68±0.13^a^	594±31^a^	108±13^a^	63.1±10.4^a^	12.13±0.52^a^
1.20	12.0±1.0^a^	1.63±0.18^a^	490±19^b^	91.7±21.6^ab^	51.8±4.5^ab^	12.12±0.51^a^
2.50	9.95±0.94^b^	1.35±0.20^b^	461±32^b^	91.7±7.1^ab^	47.9±8.7^b^	11.22±0.50^b^
3.50	6.04±0.62^c^	1.26±0.03^bc^	444±47^b^	89.2±15.5^ab^	46.0±8.4^b^	10.54±0.58^b^
4.50	5.93±0.73^c^	1.13±0.07^c^	443±29^b^	87.2±10.2^ab^	46.6±8.5^b^	10.92±0.51^b^
5.50	6.02±1.06^c^	0.87±0.07^d^	347±25^c^	70.5±21.0^b^	48.0±8.7^b^	10.88±0.25^b^

Data represent means ±S.D. of four replicates. Values within the same column with different superscripts are significantly different (*P*<0.05).

## Discussion

Soybean is a source of high-quality protein due to its relatively well-balanced composition of amino acids [59]. However, soybean is also a dietary allergic source, which threatens individuals [28]. β-conglycinin is a primary storage protein and it has been identified as one of the major allergenic proteins in soybean [8]. Thus, to define mechanisms whereby excessive soybean induces growth depression in animals; this study investigated the role of purified soybean β-conglycinin, separating its effects from the effects of other anti-nutritional factors. Firstly, the present study observed that fish fed diet with β-conglycinin exhibited poor weight gain, which was also observed in piglets [7], [8] and rats [60]. However, survival was not influenced by the dietary treatments. No reports exist concerning the effects of β-conglycinin on survival rate in fish. Similar findings were observed that replacement of dietary fishmeal with more than 41% of soy protein isolate did not influence the survival, but indeed reduced the growth performance in Pacific white shrimp (*Litopenaeus vannamei*) [61]. A reduction in feed intake was regarded as the primary factor responsible for the depressed growth observed in rats [35]. In the present study, the feed intake in the β-conglycinin group was significantly lower than that of the control group, which was in accordance with the results for rats [62]. This result suggested that the growth reduction caused by β-conglycinin was most likely attributed to the suppression of feed intake. However, there was no information referring to how β-conglycinin leads to the suppression of feed intake in fish. In rats, it has been reported that β-conglycinin peptone stimulating endogenous cholecystokinin (CCK) release suppresses food intake by inducing a feeling of satiety and by reducing gastric emptying [62]. Besides feed intake, feed utilization efficiency also influences growth performance. In the present study, β-conglycinin depressed feed efficiency, indicating that the reduction of fish growth was partly attributed to the fact that β-conglycinin decreased feed efficiency. Interestingly, when fish were administered Gln with β-conglycinin, growth performances were close to or equal to those in the control group, showing that Gln might mitigate the poor growth caused by β-conglycinin.

As an initial step in understanding how β-conglycinin reduced the growth of fish, we examined the digestive and absorptive ability of the fish. In this study, β-conglycinin alone significantly decreased the activities of trypsin, chymotrypsin, lipase, creatine kinase, Na^+^, K^+^-ATPase and alkaline phosphatase in the intestine and its enterocytes. Although no reports exist concerning the effects of β-conglycinin on digestive and absorptive enzyme activity in fish, similar findings were observed that diets containing soybeans might decrease the activity of protease in tilapia [3], alkaline phosphatase in Atlantic salmon [63] and creatine kinase in Atlantic halibut (*Hippoglossus hippoglossus*) [64]. In rats, it has been reported reported that β-conglycinin peptone in the lumen stimulating endogenous cholecystokinin (CCK) release [62] which exerts a negative-feedback regulation on pancreatic enzyme secretion. It is well known that feed intake is suppressed by a feeling of satiety and by reducing gastric emptying in rats [62]. Gastric emptying is associated with the digestive and absorptive ability. Those suggested that the depressed digestive ability in this study may be in part contributed to the suppressed feed intake, which needs further investigations. In addition, when compared with those in the control, the digestive and absorptive enzyme activities in the Gln with β-conglycinin group showed no significant differences. These results indicated that dietary Gln exerted a mitigating role against the disruption in intestinal function caused by β-conglycinin.

The reduction of digestive and absorptive capacity is usually due to poor growth of the digestive organs. The exocrine pancreas is the main site for digestive enzyme synthesis and secretion in fish [65]. The present results showed that the weight and index of the hepatopancreas decreased with β-conglycinin. However, there is a lack of information in regard to the effects of β-conglycinin on the hepatopancreas in animals. Similar results were observed in cobia (*Rachycentron canadum*) that were fed diets with soybean products [41]. In addition, the disruption of absorptive capacity in fish has been traced to the growth suppression of the intestine, which was reflected by the decrease of the intestinal length index in Atlantic salmon [49]. The present results showed that the intestinal weight, length, length index and folds height in the intestine was decreased with β-conglycinin alone. In the present *in vitro* study, β-conglycinin decreased the MTT OD values of enterocytes in a dose-dependent way. Although there are no reports concerning the effects of β-conglycinin on the growth and development of fish intestine, similar results in regard to decreased jejunum villus height induced by β-conglycinin were observed in rats [60]. Additionally, when fish were fed with Gln and β-conglycinin, the poor growth of the hepatopancreas and intestine caused by β-conglycinin was partly mitigated.

The reduced growth and development of the digestive organs may be partly due to a reduction in protein deposition. This study showed that protein content in the intestine and in its enterocytes was decreased by β-conglycinin. As mentioned above, protein syntheses are regulated by TOR signaling [10]. Interestingly, the expressions of the TOR gene in the PI, MI and DI followed similar patterns to the protein content. The patterns of 4E-BP2 mRNA levels showed opposite tendencies to those of the TOR mRNA levels in the PI and DI, indicating that β-conglycinin increased the inhibition of translation. However, this study is the first to investigate the effects of β-conglycinin on the TOR/4E-BP pathway. Thus, the underlying mechanism by which β-conglycinin affects the expression of TOR and 4E-EP genes is largely unknown and needs to be further investigated. Gln can regulate TOR activity in HeLa cells [66]. Interestingly, fish fed Gln with β-conglycinin presented a partly mitigated reduction of the TOR/4E-BP pathway caused by β-conglycinin.

The impairment of intestinal growth and function by β-conglycinin might result from a destruction of intestinal integrity. Damage to the integrity of the intestine is a common phenomenon in animals consuming soybean proteins [28]. Recently, β-conglycinin has been shown to result in intestinal epithelium damage in rats [60]. Moreover, β-conglycinin was shown to cause apoptosis and structural damage to enterocytes in mice [15]. When the cell membrane is damaged, the important metabolic enzymes, such as LDH, GOT and GPT are released from the cells [27]. Thus, the activity of LDH, GOT and GPT in the culture medium are often used to assess cell damage [15], [27]. This study showed that when the β-conglycinin level exceeded 2.5 mg/mL, the activities of LDH, GOT and GPT in the culture medium increased, indicating severe damage to the cellular membrane, which agrees with the results of the study on mouse intestinal epithelium cells [15].

The intestinal injury caused by β-conglycinin may result from inflammation. It is well known that soybean protein might cause inflammation of the distal intestine in fish [50], [63]. IL-1 and TNF-α are indicated as pro-inflammatory, whereas TGF-β is indicated as an anti-inflammatory cytokine [67]. The expression of the TNF-α gene in the β-conglycinin alone group is above the control level (Figure 1a), suggesting that β-conglycinin induced an inflammatory response in the DI of fish. It is well known that inflammation may lead to increase of water content, which may be an explanation of the decreased intestinal protein content caused by β-conglycinin. However, the expression of IL-8 gene was decreased by β-conglycinin. The reasons for these differences are largely unknown and may be partly associated with the sample period; it was reported that common carp fed SBM sampled at 0, 1, 3 and 5 weeks showed different patterns of IL-1, TNF-α and TGF-β mRNA levels in the DI [67]. However, fish fed with Gln and β-conglycinin obtained the highest values of IL-1, TNF-α and TGF-β mRNA levels. TGF-β plays an important role in the prevention of mucosal inflammation [19]. Thus, the up-regulation of TGF-β levels by Gln may be involved in protecting against β-conglycinin-induced inflammation. However, these observations need more attention and additional measurements need to be made in other intestinal compartments to explain their significance.

Intestinal inflammation may invoke a subsequent peroxidative damage due to excessive ROS production [68]. In addition, the N-glycan structure of β-conglycinin is essential for the formation of dityrosine bridges, which is a ubiquitous process primarily responsible for oxidative stress [69]. MDA is one of the most easily assayed end products of lipid peroxidation [70]. The current results showed that β-conglycinin elevated MDA content in the hepatopancreas and intestine as well as in its enterocytes, supporting the hypothesis that β-conglycinin may induce oxidative damage in these organs. It is known that the end products of lipid peroxidation may bring about protein damage [21]. Protein carbonyls (PC) are the oxidative product of amino acid residue and can be used as a biomarker of oxidative damage to protein [22]. In this study, the protein oxidations in the intestine and its enterocytes exhibited a similar trend to those of MDA. The study showed that Gln might protect fish enterocytes against H_2_O_2_-induced oxidative damage [27]. However, in this study, lipid peroxidation (MDA) and protein oxidation (PC) in the hepatopancreas and intestine of fish fed with Gln and β-conglycinin only showed reduction trends when compared with those in the β-conglycinin alone group. The reasons for this result are still unknown. It may be partly because the dose of Gln used in this study was not large enough to inhibit β-conglycinin-induced oxidative damage.

Several studies had indicated that oxidative damage was often accompanied by the reduction of antioxidant capacity, reflected by GSH content and the inhibition of antioxidant activity, including that of SOD, CAT, GPx, GST and GR [27], [44]. Therefore, in the current study, non-enzymatic and enzymatic antioxidant responses were also measured. The current study demonstrated that the content of GSH in the hepatopancreas, intestine and its enterocytes was reduced by β-conglycinin. It has been reported that the gut was one of the major sites for GSH synthesis in rats [71]. GSH synthesis in endothelial cells was through a process that required the activity of γ-GT [72]. Thus, in this study, the increase in γ-GT activity may correspond to a first attempt to overcome oxidative stress by producing a high amount of GSH. In agreement with this result, an increase of γ-GT activity was observed in the liver and white muscle of Nile tilapia exposed to ammonia [73]; however, we do not know why GSH decreased when γ-GT activity increased in this study. We hypothesize that GSH was consumed during the scavenging process for the oxidation induced by β-conglycinin. Moreover, our results showed that with dietary β-conglycinin treatment alone, the activities of SOD, CAT, GPx and GR in the hepatopancreas and intestine and the activity of intestinal GST decreased significantly when compared with those in the control group. Moreover, significant reductions in SOD, CAT, GPx, GST and GR activity were also observed in enterocytes treated with a certain level of β-conglycinin. However, in contrast to the reduced activities of these enzymes, the mRNA levels of the CuZnSOD, MnSOD, CAT and GPx1b genes increased with β-conglycinin. β-conglycinin-induced increases in the mRNA expression of antioxidant enzymes may be related to an adaptive mechanism against stress. Concurring with this hypothesis, an over-expression of cardiac-specific CAT prevents injury to the heart following oxidative damage induced by exposure to the anticancer agent doxorubicin [74]. Thus, perhaps these antioxidant enzymes were largely consumed during the defense against the oxidation caused by β-conglycinin. In addition, in the Gln with β-conglycinin group, most antioxidant enzyme activities and gene expressions are close or equal to those values in the control group. Our previous study showed that in enterocytes treated with Gln in the presence of H_2_O_2_, antioxidant enzyme activities were higher than treatment with H_2_O_2_ alone and were close to the values in the control group [27], proving that β-conglycinin damages the antioxidant system of fish.

Gln is a precursor of nucleotides, glucose, other amino acids and serves as a major fuel for intestinal epithelial cells in mammals [75]. Recent studies have demonstrated that dietary Gln supplementation could improve growth performance, intestinal structure and function in carp [24], as well as enhance the antioxidant defense in hybrid sturgeon (*Acipenser schrenckii ♀×Huso dauricus ♂*) [76]. According to these findings, we investigated the potential protection of Gln against β-conglycinin toxicity in the present study. As results, compared with the β-conglycinin alone group, dietary supplementation with Gln significantly increased SGR, feed intake, feed efficiency, hepatopancreas protein, hepatopancreas weight, intestinal protein, intestinal weight, intestinal length, intestinal length index and foregut folds height, and also significantly increased the activities of intestinal digestive enzymes including trypsin, lipase and chymotrypsin, absorptive enzymes including creatinkinase, Na^+^, K^+^-ATPase and alkaline phosphatase in the PI, MI and DI, as well as antioxidant enzymes including CAT and GR in hepatopancreas and SOD, CAT, GPx in the intestine. Moreover, SGR, intestinal length index, activities of intestinal trypsin, lipase, chymotrypsin, creatinkinase, Na^+^,K^+^-ATPase, alkaline phosphatase and GPx were restored to have no significant difference compared with the control. These results indicated that dietary Gln exerted a protective role against the growth suppression, intestinal integrity damage and function disruption induced by β-conglycinin.

### Conclusion

**Figure 2:**
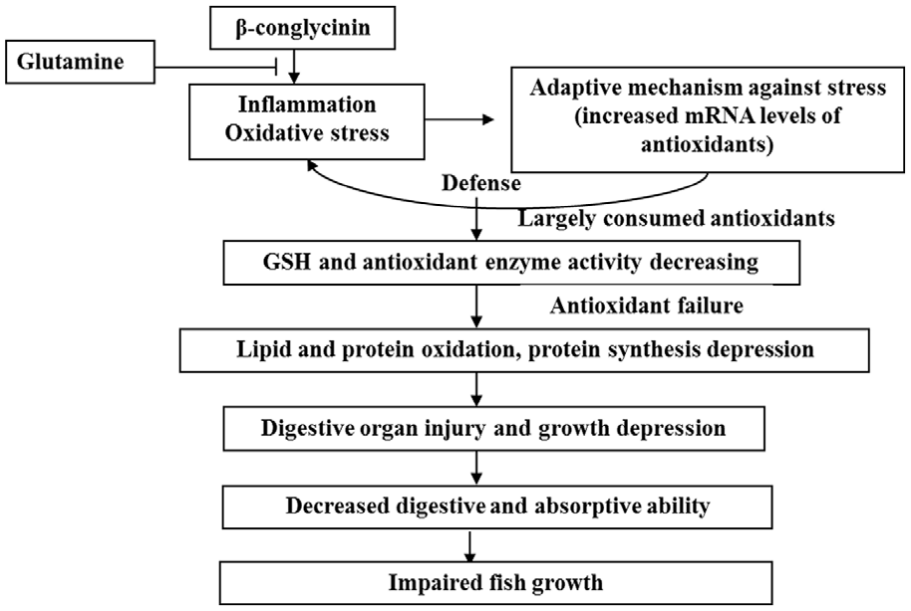
The potential action pathway of β-conglycinin-induced depression of fish growth.

The potential action pathway of β-conglycinin-induced poor growth of fish is presented in Figure 2. Briefly, this study indicates that β-conglycinin can cause oxidative damage (and impair antioxidant system in animals), and thus lead to damage and poor growth of digestive organs, subsequent by dysfunction of digestion and absorption, and finally reduce fish growth, which may provide some information to the mechanism of β-conglycinin-induced negative effects in fish. Moreover, since Gln was observed to mitigate negative influences of β-conglycinin, it is reasonable for us to recommend supplementation with Gln when high levels of SBM (β-conglycinin) are used in fish diets.

## Supporting Information

Table S1 Ingredients and nutrient content of the experimental diets (*in vivo* experiment).
(DOC)

Table S2 Real-time PCR primer sequences.
(DOC)
