# Supplementary material for: Soybean β-Conglycinin Induces Inflammation and Oxidation and Causes Dysfunction of Intestinal Digestion and Absorption in Fish
Source: PLoS One. 2013 Mar 8;8(3):e58115. doi: 10.1371/journal.pone.0058115 (PMC3592885; doi:10.1371/journal.pone.0058115)
Supplement: Table S1 — Ingredients and nutrient content of the experimental diets (in vivo experiment). (DOC) [file pone.0058115.s001.doc]

Table S1

Ingredients and nutrient content of the experimental diets (*in vivo* experiment)

| **Ingredients (g/kg)** |  |  |  |
| --- | --- | --- | --- |
| Fish meal | 125.00 | 125.00 | 125.00 |
| Gelatin | 100.00 | 100.00 | 100.00 |
| Casein | 89.60 | 89.60 | 89.60 |
| Casein | 90.40 | 0 | 0 |
| β-conglycinin | 0 | 80.00 | 80.00 |
| α-starch | 150.00 | 150.00 | 150.00 |
| Corn starch | 320.16 | 325.66 | 326.00 |
| Soybean oil | 18.00 | 18.0 | 18.0 |
| Fish oil | 18.00 | 18.00 | 18.00 |
| Ca(H_2_PO_4_)_2_ | 24.00 | 24.00 | 24.00 |
| Glutamine | 0.00 | 0.00 | 12.00 |
| L-glycin | 12.34 | 12.34 | 0.00 |
| L-lysine-hydrochloride | 0 | 2.00 | 2.00 |
| DL- methionine | 5.50 | 6.80 | 6.80 |
| L-threonine | 5.20 | 6.80 | 6.80 |
| Cellulose | 20.00 | 20.00 | 20.00 |
| Choline chloride (500 g/kg) | 1.30 | 1.30 | 1.30 |
| Trace mineral premix^2^ | 10.00 | 10.00 | 10.00 |
| Vitamin premix^3^ | 10.00 | 10.00 | 10.00 |
| Ethoxyquin (300 g/kg) | 0.50 | 0.50 | 0.50 |
| **Nutrient content (%)** |  |  |  |
| Crude protein | 33.84 | 33.97 | 33.90 |
| Crude lipid | 4.56 | 4.43 | 4.48 |
| Crude ash | 6.18 | 6.26 | 6.15 |
| Available phosphorus | 0.60 | 0.57 | 0.57 |
| Lysine | 2.19 | 2.19 | 2.19 |
| Methionine+cystine | 1.44 | 1.43 | 1.43 |
| n-3 fatty acids | 1.00 | 1.00 | 1.00 |
| n-6 fatty acids | 1.00 | 1.00 | 1.00 |

^1^Crude protein, crude lipid and crude ash content were determined according to the method of AOAC (1998). Available phosphorus, lysine, Methionine+cystine, n-3 and n-6 fatty acids contents were calculated according to NRC (1993)

^2^Per kilogram of trace mineral premix (g/kg): CuSO_4_.5H_2_O (250 g/kg copper) 1.20 g, MnSO_4_.H_2_O (318 g/kg manganese) 4.09 g, KI (38 g/kg iodine) 2.90 g, NaSeO_3_ (10 g/kg selenium) 2.50 g, ZnSO_4_.7H_2_O (225 g/kg zinc) 21.64 g, FeSo_4._7H_2_O (197 g/kg iron) 69.70 g. All ingredients were diluted with CaCO_3_ to 1 kg.

^3^Per kilogram of vitamin premix (g/kg): retinyl acetate (500,000 IU/g) 0.80 g, cholecalciferol (500,000 IU/g) 0.48 g, DL-α-tocopherol acetate (500 g/kg) 20.00 g, menadione (500 g/kg) 0.20 g, cyanocobalamin (100 g/kg) 0.01 g, D-biotin (200 g/kg) 0.50 g, folic acid (960 g/kg) 0.52 g, thiamin nitrate (980 g/kg) 0.10 g, ascorhyl acetate (920 g/kg) 7.24 g, niacin (980 g/kg) 2.85 g, meso-inositol (980 g/kg) 52.86 g, calcium-D- pantothenate (980 g/kg) 2.51 g, riboflavine (800 g/kg) 0.63 g, pyridoxine hydrochloride (980 g/kg) 0.76 g. All ingredients were diluted with corn starch to 1 kg.
