# Supplementary material for: Soybean β-Conglycinin Induces Inflammation and Oxidation and Causes Dysfunction of Intestinal Digestion and Absorption in Fish
Source: PLoS One. 2013 Mar 8;8(3):e58115. doi: 10.1371/journal.pone.0058115 (PMC3592885; doi:10.1371/journal.pone.0058115)
Supplement: Table S2 — Real-time PCR primer sequences. (DOC) [file pone.0058115.s002.doc]

Table S2

**Real-time PCR primer sequences**

| Name | Sequences of Primers | Gene Bank No. |
| --- | --- | --- |
| **TOR** |  | **FJ899680** |
| forward | 5’-ATCATACGCATCCAGTCCATTG-3’ |  |
| reverse | 5’-GGTCATTAGCCAGTAGAGTGTTC-3’ |  |
| **4E-BP** |  | **HQ010440** |
| forward | 5’-GCTACCTCACGACTATTGC-3’ |  |
| reverse | 5’-TTCTTGCTTGTCACTCCTG-3’ |  |
| **IL-1** |  | **AB010701.1** |
| forward | 5’- AACTTCACACTTGAGGAT -3’ |  |
| reverse | 5’- GACAGAACAATAACAACAAC -3’ |  |
| **TNF-α** |  | **AJ311800.2** |
| forward | 5’- AAGTCTCAGAACAATCAGGAA -3’ |  |
| reverse | 5’- TGCCTTGGAAGTGACATT -3’ |  |
| **TGF-β** |  | **U66874.1** |
| forward | 5’- CAGCACAGCAATATCCTT -3’ |  |
| reverse | 5’- GTTCCAGATCCTGAGACA -3’ |  |
| **IL-8** |  | **DQ453125.1** |
| forward | 5’- TGAAGGAATGAGTCTTAG -3’ |  |
| reverse | 5’- CAATGATCTCTGTGTCTT -3’ |  |
| **CuZnSOD** |  | **JF342355** |
| forward | 5’- TGGCGAAGAAGGCTGTTTGT -3’ |  |
| reverse | 5’- TTCACTGGAGACCCGTCACT -3’ |  |
| **MnSOD** |  | **JF411603** |
| forward | 5’- CTGCCTGACCTTCCATACGA -3’ |  |
| reverse | 5’- CCTTAGCCAGTGCCTCTTGATA -3’ |  |
| **CAT** |  | **JF411604** |
| forward | 5’- CTGGAAGTGGAATCCGTTTG -3’ |  |
| reverse | 5’- CGACCTCAGCGAAATAGTTG -3’ |  |
| **GPX1a** |  | **JF411605** |
| forward | 5’- GTGACGACTCTGTGTCCTTG -3’ |  |
| reverse | 5’- AACCTTCTGCTGTATCTCTTGA -3’ |  |
| **GPX1b** |  | **JF411606** |
| forward | 5’- TATGTCCGTCCTGGCAATGG -3’ |  |
| reverse | 5’- ATCGCTCGGGAATGGAAGTT -3’ |  |
| **GR** |  | **JF411607** |
| forward | 5’- GAGAAGTACGACACCATCCA -3’ |  |
| reverse | 5’- CACACCTATTGAACTGAGATTGAG -3’ |  |
| **β-actin** |  | **M24113** |
| forward | 5’-CGTGATGGACTCTGGTGATG-3’ |  |
| reverse | 5’-TCGGCTGTGGTGGTGAAG-3’ |  |
